# Supplementary material for: CD32 expression is associated to T-cell activation and is not a marker of the HIV-1 reservoir
Source: Nat Commun. 2018 Jul 16;9:2739. doi: 10.1038/s41467-018-05157-w (PMC6048139; doi:10.1038/s41467-018-05157-w)
Supplement: Supplementary file 1 — Supplementary Information [file 41467_2018_5157_MOESM1_ESM.pdf]

Supplementary Fig. 1

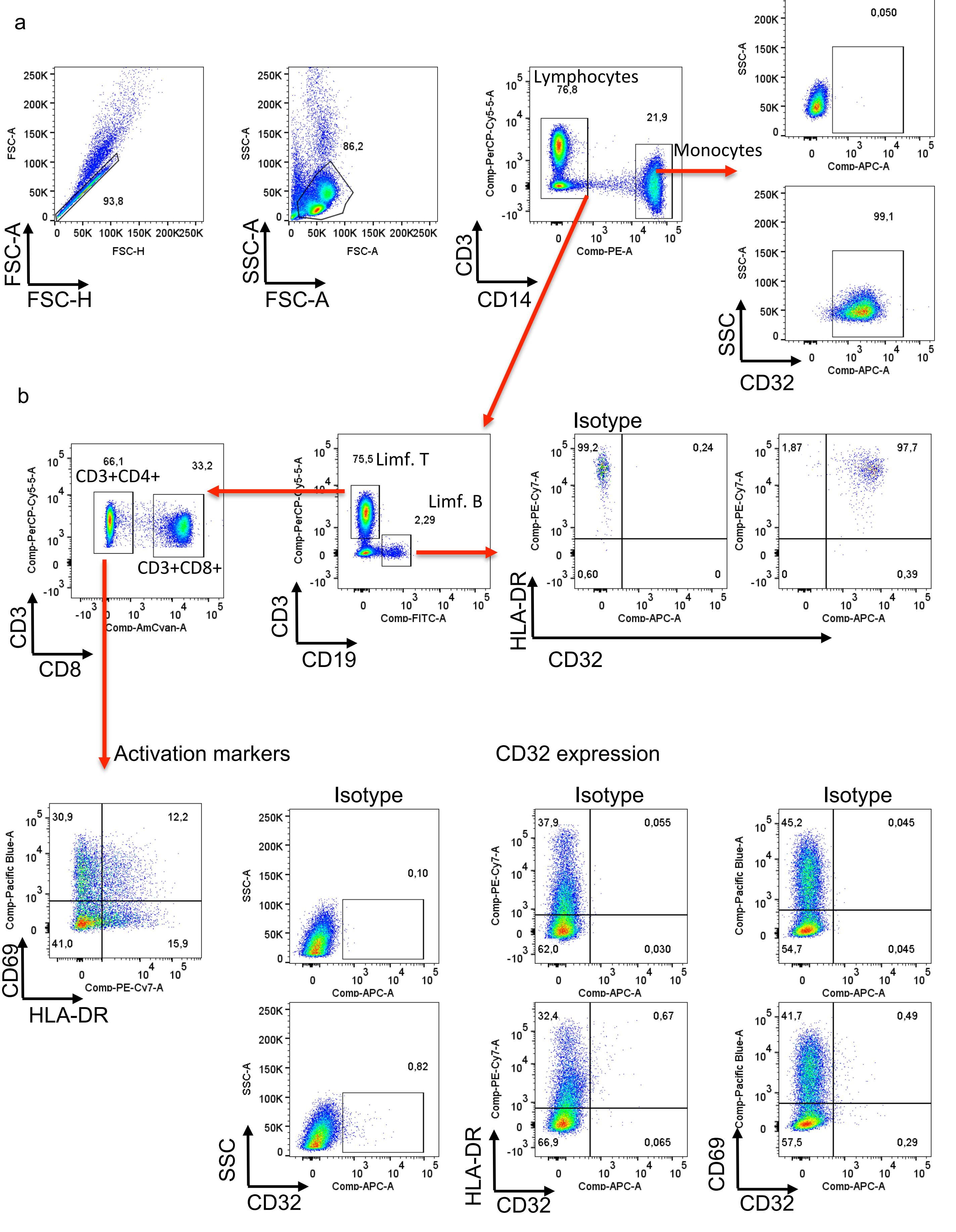

Supplementary Figure 1. Gating strategy in the IL-2, PHA/IL-2 and α-CD3/α-CD28 treated PBMCs or CD4+ T cells from donors. (a) Cell doublets were removed from the analysis (FSC-A versus FSC-H) and lymphocytes were gated by using the forward and side scatter areas (FSC and SSC). Monocytes (upper/right) and B lymphocytes (b) were excluded by labeling CD14 and CD19 cell surface markers. The marginal CD8+ T cell population found after negative selection was excluded and cell activation markers (HLA-DR and CD69) were measured in the CD4+ population in combination with CD32. Dot plots from a representative donor are shown.

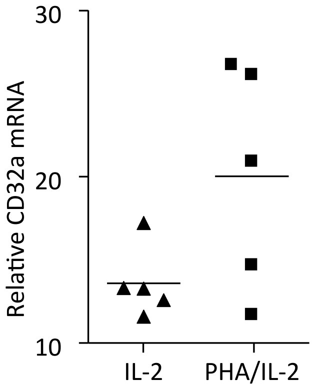

Supplementary Figure 2. mRNA expression of FGCR2a (CD32a) in stimulated CD4+ T cells. CD32 gene expression levels in purified CD4+ T cells after activation with IL-2 or PHA and IL-2. Relative mRNA expression of FGCR2a was measured using quantitative PCR and normalized to GAPDH expression. The data represent 1/ΔCt*100.

Supplementary Figure 3. Purity of CD32+ cells in the CD4+ T compartment. (a) Contamination of monocytes or lymphocytes B was discarded by evaluation of CD14+ and CD19+ cell surface expression in both CD4+ T cells and in CD32+ CD4+ T cells samples from five different donors. (b) Percentage of CD32 cell surface expression in CD4+ T lymphocytes, B cells and monocytes from PBMCs from five different donors (c) and 17-20 HIV-1 infected individuals. Student's t test, *** p<0.0001

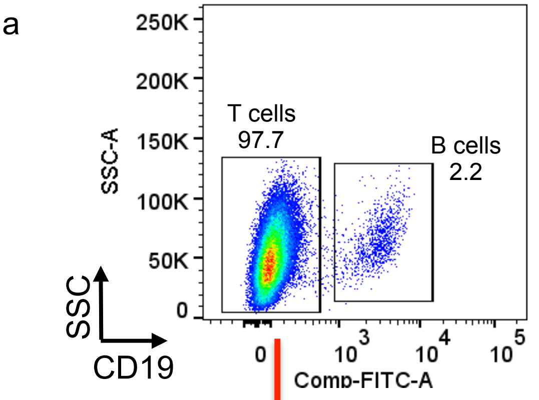

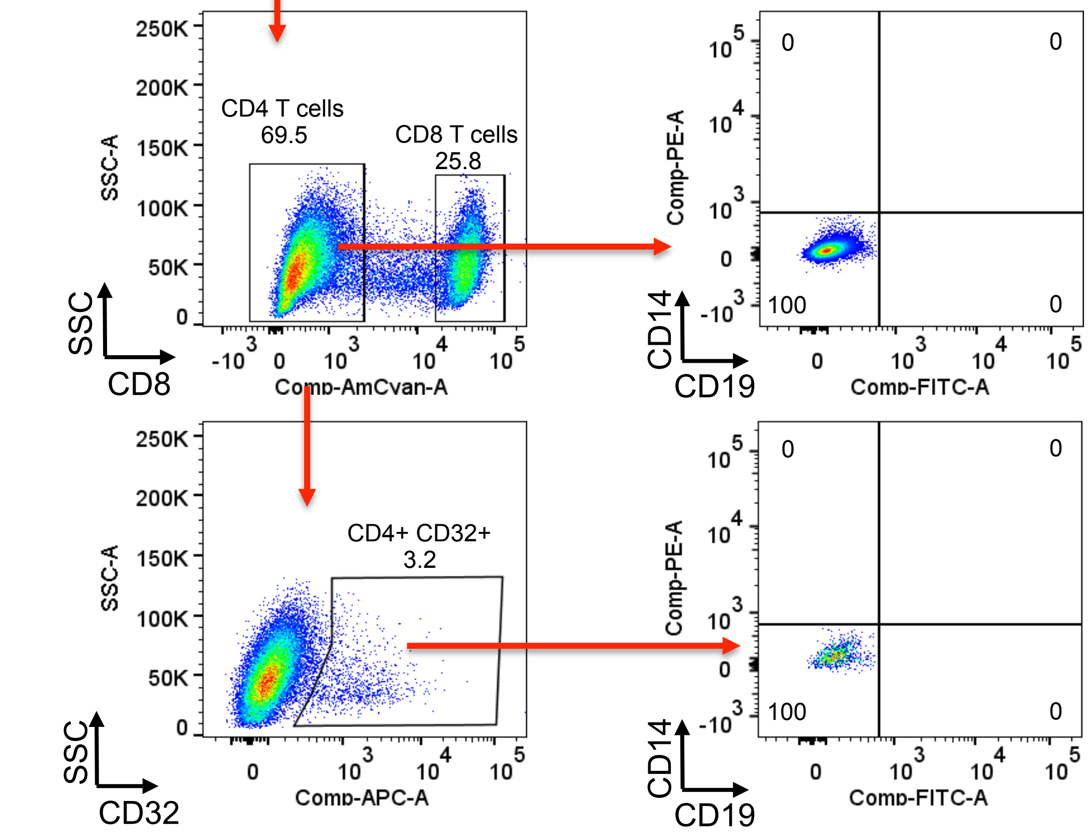

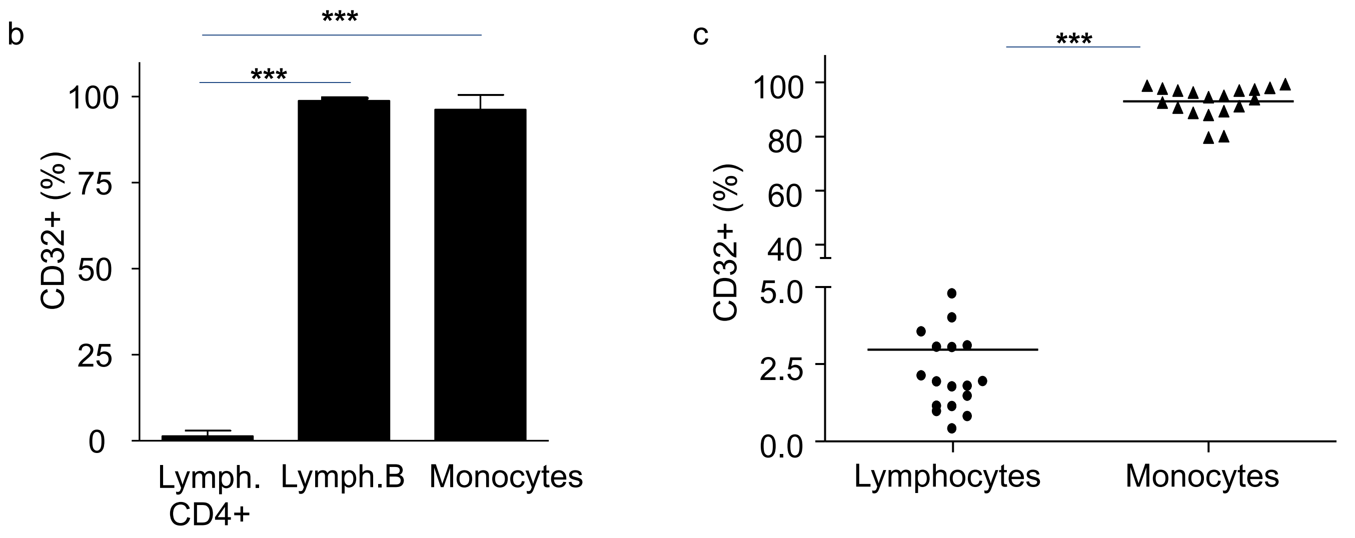

Supplementary Figure 4. Gating strategy in the PBMCs from HIV-1 infected individuals. Cell doublets were removed from the analysis (FSC-A versus FSC-H) and PBMCs were gated by using the forward and side scatter areas (FSC and SSC). As most of CD32+ cells are CD14+, CD14 was stained and excluded from further analysis. Then, the CD4+ compartment was selected by CD8+ exclusion and CD32 was evaluated in combination with cell activation markers (HLA-DR and CD69). Dot plots from a representative participant are shown.

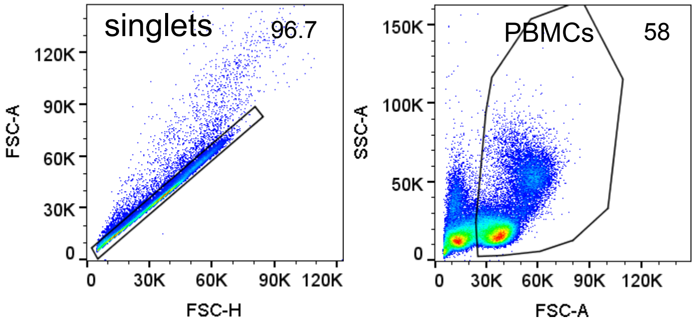

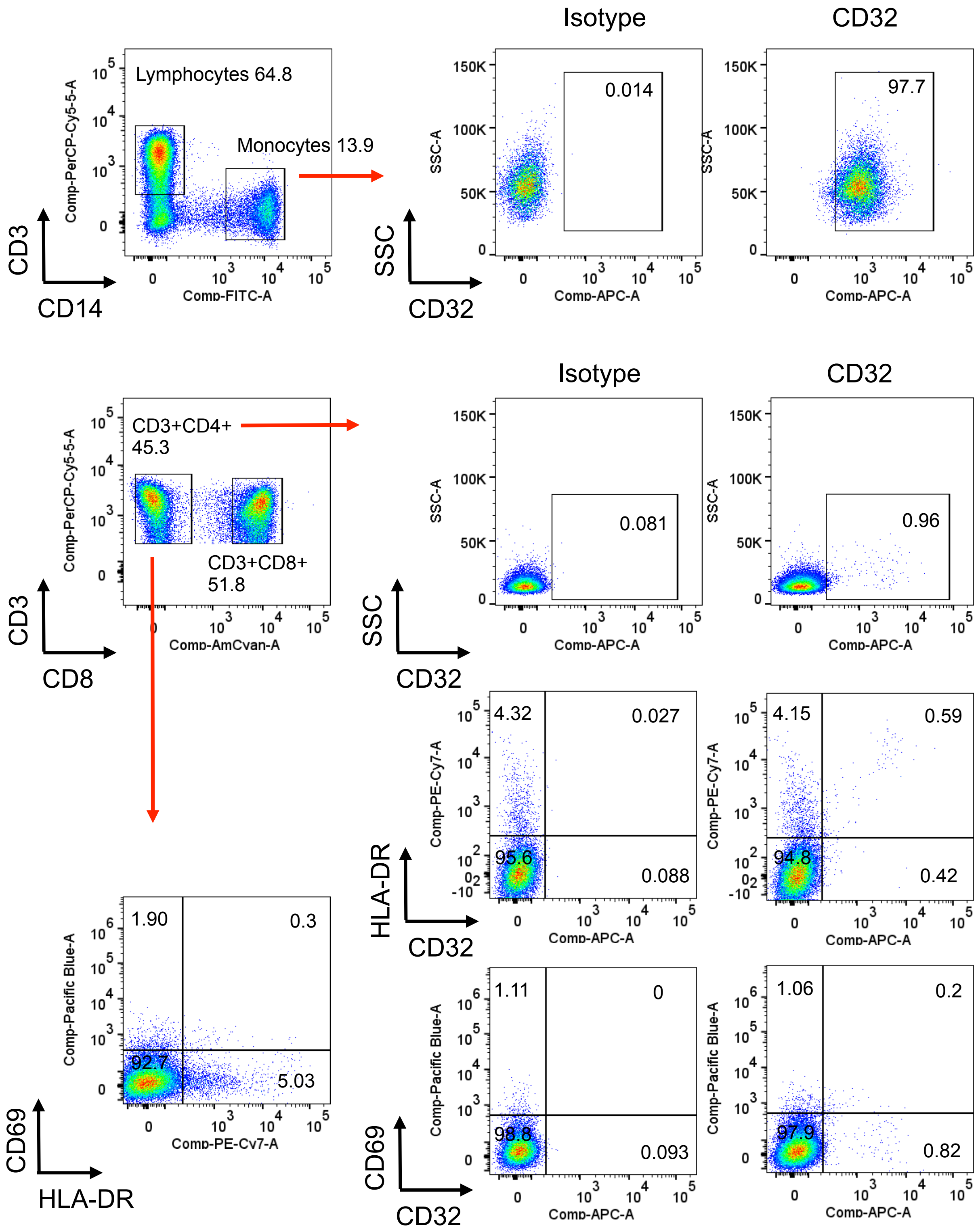

Supplementary Figure 5.

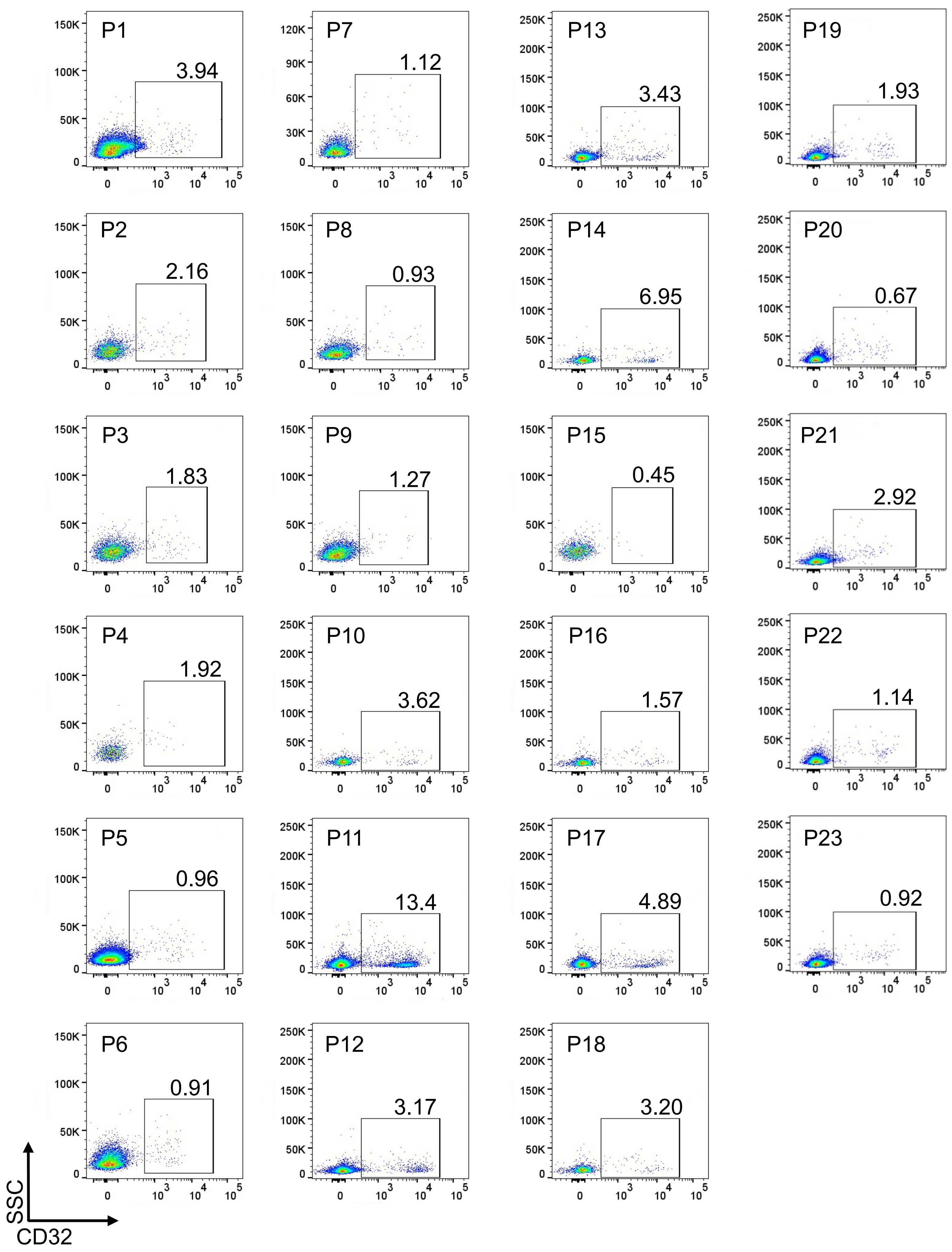

Supplementary Figure 5. CD32 staining in HIV-1 infected individuals. Individual dot plots from the 23 HIV-1+ subjects evaluated, showing CD32 staining in previously gated CD4+ T cells. Individual isotype control labeling was set to a stringent criteria (less or equal to 0.1% positive cells).
